# In planta expression of active bacterial GDP‐6‐deoxy‐d‐lyxo‐4‐hexulose reductase for glycan modulation

**DOI:** 10.1111/pbi.14131

**Published:** 2023-08-08

**Authors:** Benjamin Kogelmann, Roman Palt, Daniel Maresch, Richard Strasser, Friedrich Altmann, Somanath Kallolimath, Lin Sun, Marc‐André D'Aoust, Pierre‐Olivier Lavoie, Pooja Saxena, Johannes S. Gach, Herta Steinkellner

**Affiliations:** ^1^ Department of Applied Genetics and Cell Biology University of Natural Resources and Life Sciences Vienna Austria; ^2^ ACIB – Austrian Centre of Industrial Biotechnology Vienna Austria; ^3^ Core Facility Mass Spectrometry University of Natural Resources and Life Sciences Vienna Austria; ^4^ Department of Chemistry University of Natural Resources and Life Sciences Vienna Austria; ^5^ Medicago Inc. Quebec QC Canada; ^6^ University of California Division of Infectious Diseases Irvine CA USA

**Keywords:** RMD, N‐glycan engineering, fucose, *Nicotiana benthamiana*, recombinant glycoproteins

Plant‐produced glycoproteins carry α1,3‐linked core fucosylated N‐glycans, and the reduction/elimination thereof often confers beneficial features (Strasser *et al*., 2008; Zeitlin *et al*., 2011). Here, we evaluated the possibility of altering fucosylation *in planta* by overexpressing a bacterial GDP‐6‐deoxy‐d‐lyxo‐4‐hexulose reductase (RMD), an enzyme negatively interfering with the GDP‐l‐fucose biosynthesis pathway (von Horsten *et al*., 2010).

RMD gene from *Pseudomonas aeruginosa* (Figure S1) was transiently expressed in *Nicotiana benthamiana* wild‐type (WT) plants (Figure S2). MS‐based N‐glycosylation profiles from total soluble proteins (TSP) showed that overall fucosylation decreased from 64% to 36% compared to TSP lacking RMD (Figure 1a) while leaving the overall N‐glycan composition largely unchanged. MALDI‐TOF MS peaks of fucosylated glycans were accompanied by peaks which, at a cursory glance, could have been interpreted as potassium adduct ions. A more consistent interpretation, however, identifies these peaks as consisting mainly of glycans that contain hexose (possibly l‐galactose) instead of fucose.

**Figure 1:**
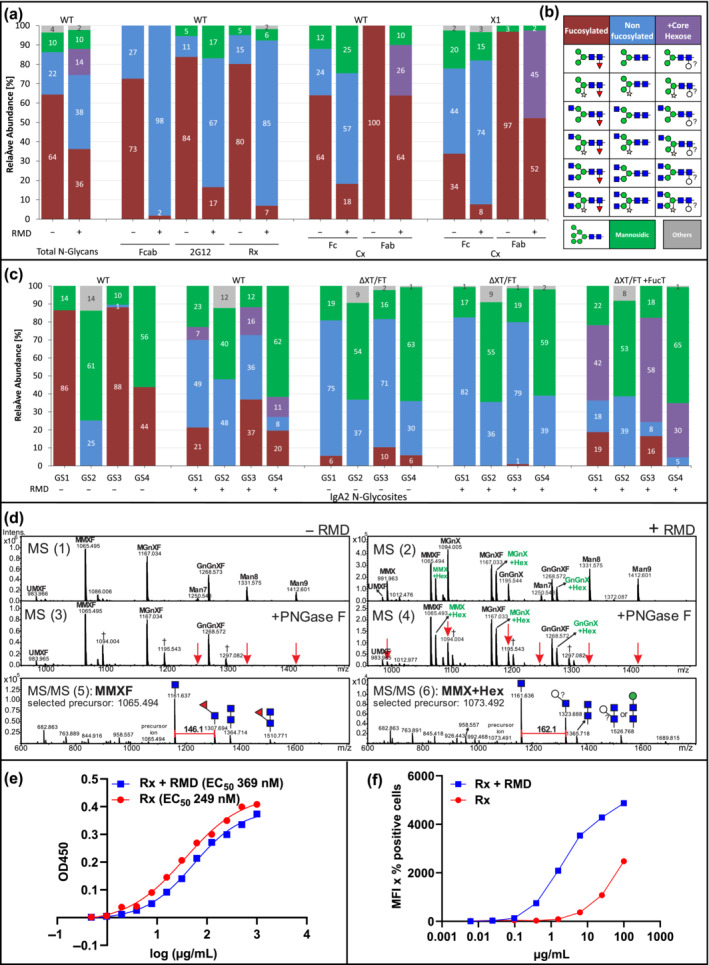
(a) N‐glycan profiles of *Nicotiana benthamiana* total soluble proteins (TSP) and IgG1 reporter with (+) or without (−) RMD. Rituximab (Rx), cetuximab (Cx), 2G12, and Fcab (Fc fragment) were used. WT: wild type, X1: XylT‐RNAi mutant (Strasser *et al*., 2008). (b) Schematic presentation of glycans; according to the Consortium for Functional Glycomics (www.functionalglycomics.org). (c) N‐glycan profiles of IgA2 expressed in *N. benthamiana* WT or ΔXT/FT (Strasser *et al*., 2008) with (+) or without (−) RMD. Individual glycosylation sites (GS) are numbered. Bars represent the relative abundance (%) of detected glycoforms (see also Tables S2 and S3). (d) WT produced IgA2 GS3 N‐glycans. MS spectra (1, 3): (−) RMD; (2, 4): (+) RMD; (3, 4) + PNGase F digestion. The additional peaks shifted by ~162 Da (mass of a hexose are indicated in green letters). MS/MS profiles (5, 6) representing collision‐induced fragmentation of IgA2 GS3 MMX structures − /+ RMD, respectively. Red arrows: PNGase F digested structures. Grey cross: N‐terminal iodacetamide (Carbamidomethylation, CAM, artefacts, +57.02 Da), only emerging after heat treatment (for trypsin inactivation) present in (3) and (4); 1094.004, 1195.543 and 1297.082 represent CAM of MMXF (1065.494), MGnXF (1167.034) and GnGnXF (1288.574), respectively. The mass difference between adding a GlcNAc residue (+203.08 Da) and removal of fucose (−146.06 Da) has the same mass difference as CAM (+57.02 Da). Masses in the MS spectra are [M + 2H]2+ ions. (e) Antigen binding ELISA of Rx (Rx + RMD). (f) Binding affinity of Rx and Rx + RMD against the TZM‐bl FcγRIIIa receptor.

Next, different monoclonal antibodies (Abs; Rx, rituximab, Cx, cetuximab, 2G12 and an Fc‐fragment, Fcab) were transiently expressed (Figure S3; Castilho *et al*., 2015; Li *et al*., 2016; Strasser *et al*., 2008) and subjected to glycopeptide‐analyses. The Fc‐N‐glycan composition of all mAbs expressed without RMD carried mainly complex N‐glycans decorated with core xylose and fucose (Figure 1a), ranging from 70% to 90%. Upon RMD expression, the amounts of fucosylated N‐glycans were significantly reduced with otherwise only minor changes in the N‐glycan profile (Figure 1a,b). The most prominent fucose reduction was observed for Fcab (from 70% to <2%). Cx, which carries two GSs, was also expressed in *N. benthamiana* X1, a xylosyltransferase RNAi line synthesizing complex N‐glycans largely devoid of β1,2‐xylose (Strasser *et al*., 2008). Both Cx GSs exhibited significantly reduced fucosylated N‐glycans upon RMD expression, from 40% to 10% and from 97% to 50%, at Fc and Fab, respectively (Figure 1a,b).

Next, IgA2 an Ab isotype that carries four GSs (GS 1–4 from N‐ to C‐ terminus) was expressed in WT *N. benthamiana* plants (Figure S3). GS1 and GS3 carried 85%–90% fucosylated N‐glycans, whereas GS4 exhibited only about 40% thereof and GS2 N‐glycans lack fucose (Figure 1c). Upon RMD co‐expression, a significant reduction of fucose‐carrying N‐glycans was observed at GS1, 3 and 4, while at the same time, overall glycosylation was largely unchanged. Interestingly, upon RMD expression MS spectra revealed extra peaks that were not present in the absence of RMD, especially at GS1, GS3 and GS4 (Figure 1c) and on the Fab N‐glycan from cetuximab (Figure 1b). Some peaks were accompanied by masses of additional ~162 Da, suggesting the attachment of a hexose (e.g. galactose) instead of fucose (146 Da). To investigate if the attachment of this additional hexose is dependent on the α1,3‐fucosyltransferase, IgA2 was expressed in *N. benthamiana* ΔXT/FT background (RNAi downregulated fucosyl/xylosyltransferase), that synthesize complex N‐glycans largely devoid of xylose and fucose (Strasser *et al*., 2008). ΔXT/FT‐derived IgA2 exhibits complex N‐glycans largely devoid of plant‐specific residues and co‐expression of RMD further pronounced fucose reduction (Figure 1c). In contrast, by co‐expressing a plant α1,3 fucosyltransferase (Castilho *et al*., 2015) and RMD in ΔXT/FT, glycans with the additional 162 Da peak appeared (up to 59%), indicating a fucosyltransferase‐mediated attachment.

PNGase F treatment was performed to analyse these RMD‐associated peaks (Figure 1d). The enzyme digested structures without core‐GlcNAc modification (i.e., oligomannosidic glycans, MGnX, GnGnX and MMX), whereas glycan peaks (assigned as MMX + Hex, MGnX+Hex, GnGnX+Hex) remained unmodified. These results indicate the attachment of a hexose at the core GlcNAc thereby blocking PNGase F activity (Figure 1d). The peaks with the highest intensity (i.e. MMXF and MMX + Hex) were subjected to collision‐induced fragmentation (MS/MS, Figure 1d). The respective MMXF profile exhibited a peak representing a peptide carrying GlcNAc and fucose (+146.1 Da), as expected for core fucosylated N‐glycans. However, upon RMD co‐expression, the MMX + Hex peak was fragmented in a peptide with a GlcNAc carrying an additional hexose (+162.1 Da, Figure 1d), suggesting the addition of a core hexose (most probably galactose) to the core GlcNAc residue. Notably, the additional hexose was not detected at all glycosites. Similar differential behaviour was observed for ΔXTFT RNAi knock‐down plants, where the remaining low levels of FucT sufficed to add some fucose to Fab glycans, while Fc glycans remained essentially unfucosylated (Castilho *et al*., 2015).

Finally, functional activities were determined for Rx expressed in WT plants with and without RMD. Similar antigen binding of both Ab variants was observed (Figure 1e). In contrast, compared to Rx, Rx + RMD exhibited increased binding to cellular receptor FcγRIIIa (Figure 1f), as expected for IgG1 Abs with reduced core fucosylation. The experiments show no functional impairments upon RMD expression.

Collectively, we demonstrate the expression of a functionally active bacterial RMD in plants. Notably, our data suggest, that suppressed availability of GDP‐l‐fucose allows the core α1,3‐fucosyltransferase to use the structurally related UDP‐l‐galactose as donor substrate, in line with other results that report the transfer of l‐galactose to N‐glycans in case of GDP‐l‐fucose shortage (Ohashi *et al*., 2017; Rayon *et al*., 1999). This should be considered by RMD‐based glycan engineering for commercial purposes (Puklowski *et al*., 2013). In summary, transient co‐delivery of bacterial RMD into WT plants provides a straightforward alternative for the removal of core fucose compared to laborious genome editing approaches (Jansing *et al*., 2019).

## Conflict of interests

The authors declare no conflicts of interest.

## Author contributions

HS, BK, SK, RS, FA, JG, and LS designed research; BK, RP, DM, JG, and LS performed experiments; all contributed to data interpretation and manuscript writing.

## Supporting information


**Appendix S1** Material and methods.
**Figure S1** Schematics of RMD expression construct.
**Figure S2** Heterologous expression of RMD in *N. benthamiana*.
**Figure S3** Schematic presentation of reporter glycoproteins.
**Table S1** Glycopeptides after enzymatic digest.

**Table S2** Quantified glyco‐profile of TSP and purified antibodies.
**Table S3** Quantified glyco‐profile of IgA2m1.
