# Supplementary material for: In planta expression of active bacterial GDP‐6‐deoxy‐d‐lyxo‐4‐hexulose reductase for glycan modulation
Source: Plant Biotechnol J. 2023 Aug 8;21(10):1929–31. doi: 10.1111/pbi.14131 (PMC10502745; doi:10.1111/pbi.14131)
Supplement: Supplementary file 1 — Appendix S1 Material and methods. Figure S1 Schematics of RMD expression construct. Figure S2 Heterologous expression of RMD in N. benthamiana. Figure S3 Schematic presentation of reporter glycoproteins. Table S1 Glycopeptides after enzymatic digest. [file PBI-21-1929-s001.docx]

**Supporting information**

In planta expression of active bacterial GDP-6-deoxy-D-lyxo-4-hexulose reductase for glycan modulation

Benjamin Kogelmann^a, b^, Roman Palt^a^, Daniel Maresch^c^, Richard Strasser^a^, Friedrich Altmann^d^, Somanath Kallolimath^a^, Lin Sun^a^, Marc-André D'Aoust^e^, Pierre-Olivier Lavoie^e^, Pooja Saxena^e^, Johannes S. Gach^f^, and Herta Steinkellner^a,*^

^a^ Department of Applied Genetics and Cell Biology, University of Natural Resources and Life Sciences, Vienna, Austria

^b^ acib - Austrian Centre of Industrial Biotechnology, Muthgasse 18, 1190 Vienna, Austria.

^c^ Core Facility Mass Spectrometry, University of Natural Resources and Life Sciences, Vienna, Austria

^d^ Department of Chemistry, University of Natural Resources and Life Sciences, Vienna, Austria

^e^ Medicago Inc., Quebec, QC, Canada

^f^ University of California, Irvine, Division of Infectious Diseases, Irvine, CA,

*Correspondence:

Herta Steinkellner

Department of Applied Genetics and Cell Biology, University of Natural Resources and Life Sciences, Vienna, Muthgasse 18, 1190 Vienna, Austria

Tel: +43-1-47654-94370,

Fax: +43-1-47654-94009,

**Email:** [herta.steinkellner](mailto:herta.steinkellner)@boku.ac.at

**Appendix S1 Material and Methods**

Expression constructs

A transient pCambia-based vector system was used (http://cambia.org/) for *in-planta* expression of *Pseudomonas aeruginosa* (PAO1) oxidoreductase GDP-6-deoxy-D-lyxo-4-hexulose reductase gene (RMD, GenBank: AAG08839.1) (**Figure S*1***) mediated via *A.* tumefaciens (strain GV3101).


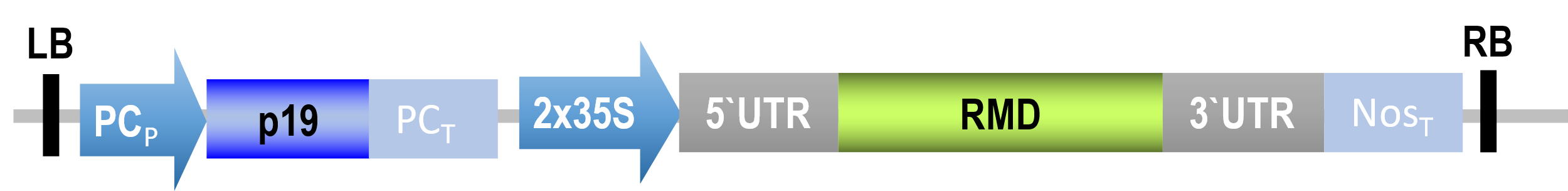


**Figure S1** Schematics of RMD expression construct. RB, LB: right and left border sequences. 2x35S: two times 35S promotor of Cauliflower Mosaic Virus, (CaMV), 5´and 3´UTR: RNA-2 Cowpea mosaic virus (CPMV) untranslated regions, RMD: GDP-6-deoxy-D-lyxo-4-hexulose reductase gene (P. aeruginosa), NosT: Nopaline synthase gene terminator (A. tumefaciens), p19: RNA silencing suppressor from tomato bushy stunt virus (TBSV); PC-P, PC-T: promoter and terminator sequence of plastocyanin gene (M. sativa).


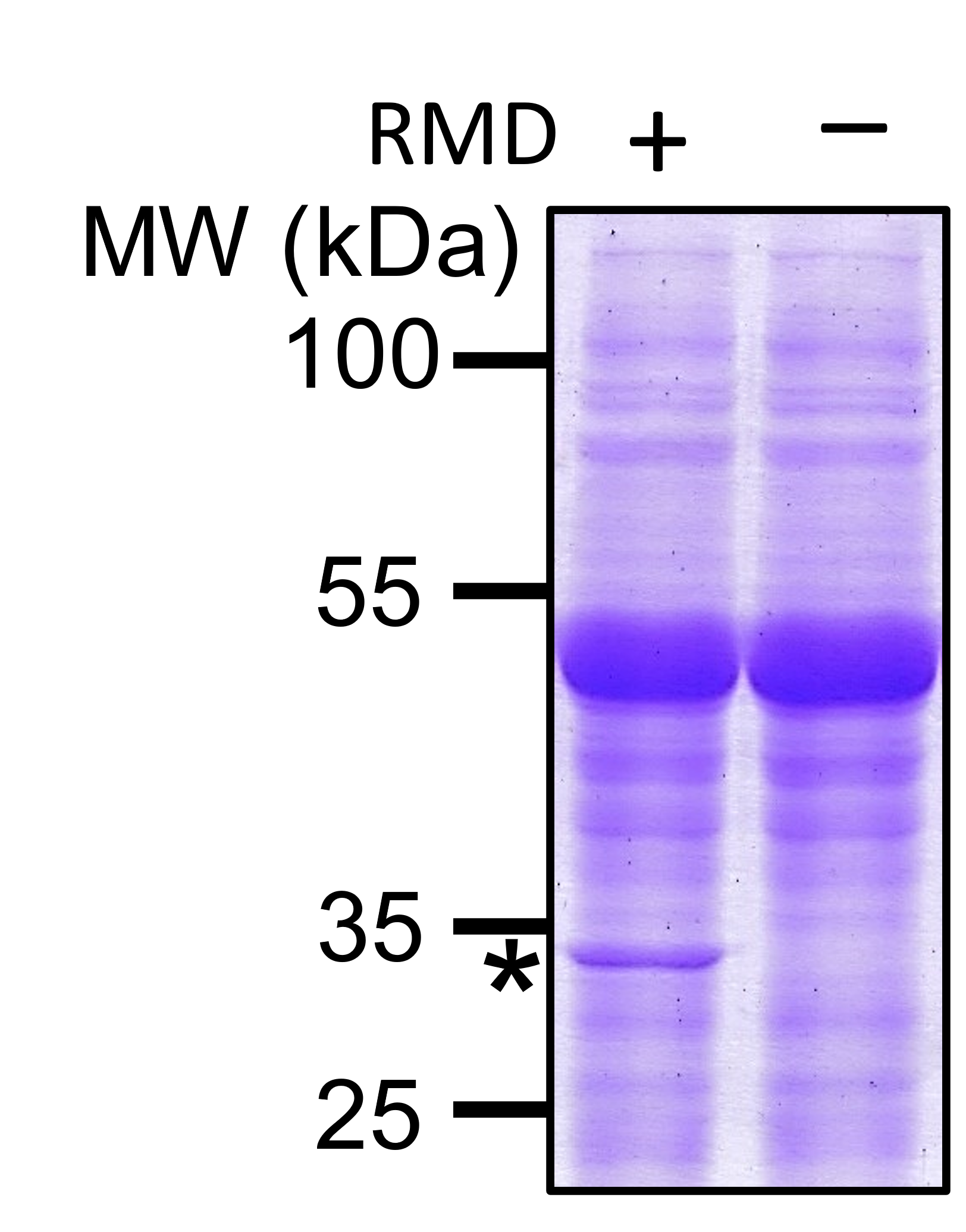


**Figure S2** Heterologous expression of RMD in *N. benthamiana*. SDS PAGE (CBB stained) of total soluble proteins (TSP). Asterisks indicates the recombinant protein (molecular mass 34 kDa).

Peptide mapping

The observed band at 34 kDa from total soluble proteins (TSP) was subjected to peptide mapping. A database search against the target proteome (https://www.uniprot.org/proteomes/UP000002438) combined with the proteome of *N. bentamiana* (in house database) confirmed the protein (RMD_PSEAE_GDP-6-deoxy-D-mannose_reductase) as the top hit.

Heterologous expressed proteins

For the expression of glycoprotein reporters different transient based vector approaches were used. All reporter were successfully expressed recently: Fcab (expected molecular mass 25 kDa) (Jez et al., 2012), monoclonal IgG1 antibodies cetuximab (Castilho et al., 2015), rituximab (Li et al., 2016), 2G12 (Strasser et al., 2008), also IgA2m1 (Goritzer et al., 2017) (expected mass of heavy and light chain: ~ 55 and ~ 30 kDa).

A hybrid fucosyltransferase (FucT) with catalytic domain of *Zea mays* core α1,3-fucosyltransferase and cytoplasmic tail, transmembrane domain and stem (CTS) region of *Arabidopsis thaliana* core α1,3-fucosyltransferase was used (Castilho et al., 2015).

Transient expression in in *N. benthamiana*

*A. tumefaciens* carrying respective recombinant plasmids were used for leaf-infiltration of 4-5 weeks old *N. benthamiana* wildtype (WT), a β1,2-xylosyltransferase RNAi knockdown X1 and a β1,2-xylosyltransferase and α1,3-fucosyltransferase RNAi knockdown ΔXT/FT, respectively (Strasser et al., 2008). Plants were grown under long-day conditions (16 h light/ 8 h dark) at 25 °C in 60% humidity. Agrobacteria cultures were grown in LB medium (5 g/L Yeast extract, 10 g/L NaCl, Tryptone/Peptone 10 g/L, pH 7.0 supplemented with respective antibiotics) overnight at 29 °C shaking at 180 rpm. The bacterial culture was pelleted (2000 x g, 10 minutes) and resuspended in infiltration buffer (10 mM MES-NaOH pH 5.6, 10 mM MgSO_4_) to set the OD_600_ to 0.05.

Extraction of total soluble proteins (TSP)

300 mg infiltrated leaf tissue was harvested between 3 to 10 dpi, frozen in liquid nitrogen and ground in a mixer mill (MM400 Retsch) with metal beads at a frequency of 30 Hz for 2 minutes. For extraction of TSP the ground leave tissue was resuspended in a 1:2 ratio w/v with TSP extraction buffer (0.1 M Tris, 0.5 M NaCl, 1 mM EDTA, 40 mM Ascorbic acid, pH 6.8-7.4). After centrifugation (13000 x g for 10 minutes), the supernatant was used for SDS-PAGE or affinity purification.

Antibody purification

Recombinant target proteins were affinity purified from TSP extracts. IgGs were purified using ProA rProteinA Agarose Resin Fast Flow (Amicogen), IgAs were purified using Capture select IgA affinity Matrix (Thermo Scientific). Samples were incubated with 30 µL of three times phosphate-buffered saline -washed (PBS, 137 mM NaCl, 2.7 mM KCl, 10 mM Na_2_HPO_4_, 1.8 mM KH_2_PO_4_, pH 7.4) resin. After incubation on a rotating mixer at 4 °C for 90 minutes, samples were centrifuged for 400 x g for five minutes. In PBS resuspended resin was loaded on a Micro Bio-Spin column (Biorad) and washed four times with PBS. Proteins were eluted with 30 µL of elution buffer (100 mM Glycine, pH 2.5) followed by neutralization to pH 7.0 using counter buffer (1 M Tris-NaOH, pH 9.0).

SDS-PAGE

~4 µg of protein samples or 12 µL of TSP were incubated in reducing conditions (Lämmli buffer) at 95 °C for 5 minutes and separated on a 12% SDS PAGE followed by Coomassie Brilliant Blue (R 250) staining.


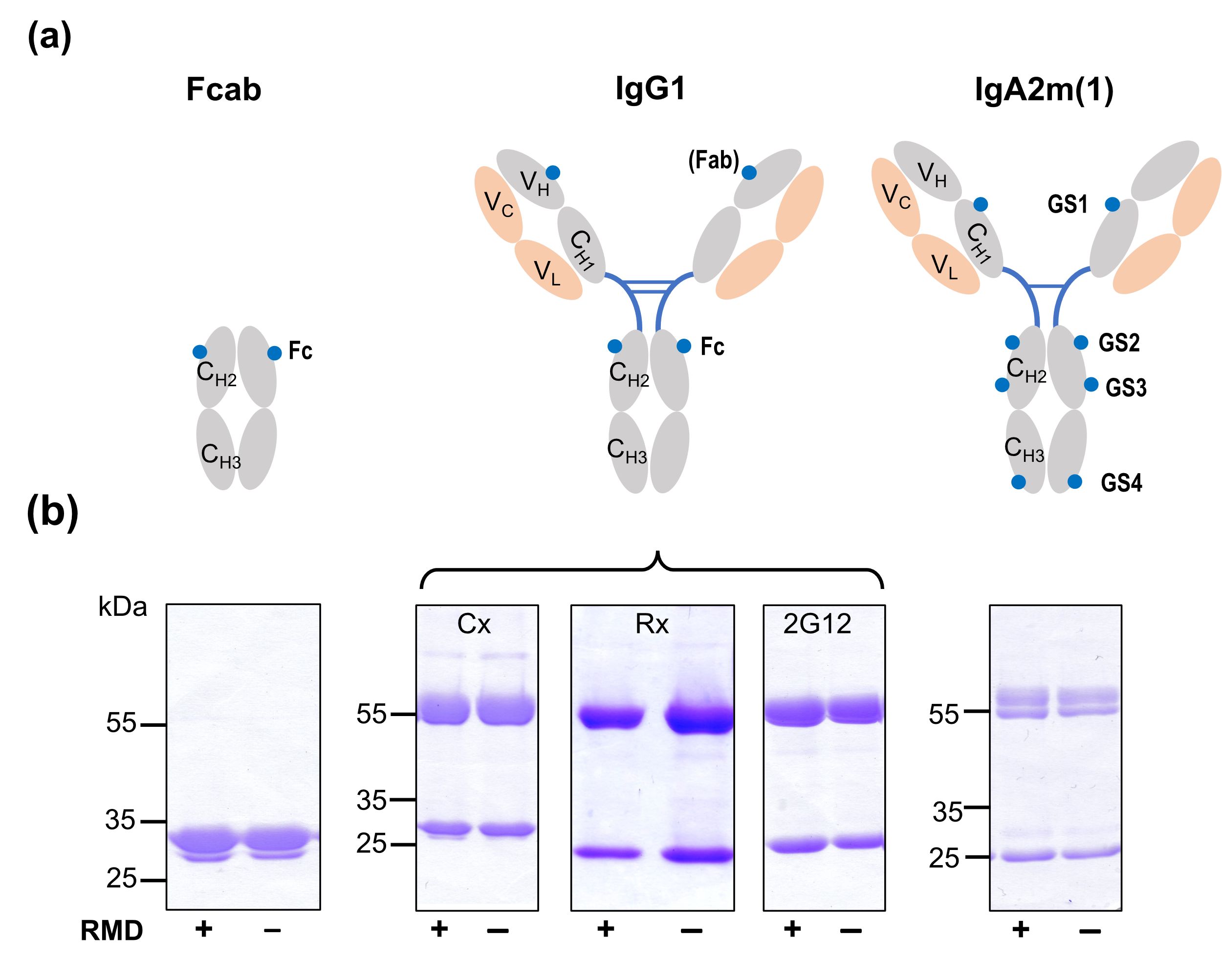


**Figure S3 (a):** Schematic presentation of reporter glycoproteins. Blue dots indicate N-glycosylation sites (GS, ascending numbering from N-C-terminus); Fcab: IgG1 Fc-fragment; IgG1 and IgA2m (1) represent different monoclonal antibodies: CH, VH: constant and variable heavy chain; CL, VL: constant and variable light chain; Fab: fragment antigen binding; **(b):** SDS-PAGE (CBB stained) of purified recombinant proteins expressed with or without RMD (+/- RMD). Side bars represent molecular mass (in kDa), Cx: cetuximab, Rx: rituximab; 2G12: anti-HIV. Note: smaller band of IgA HC (~55 kDa) represents the heavy chain lacking the tail piece.

In-gel digest

For glycan analysis of IgG, samples were subjected to an in-gel digest. Specific bands (i.e. heavy chain) were extracted from the gel, washed 2 times with 50 µL of 50% acetonitrile for 15 minutes and once with 50 µL 100% acetonitrile for 30 seconds. After two incubation steps in 30 µL of 0.1 M NH_4_HCO_3_ for 5 minutes at room temperature (RT) and 30 µL of 100% acetonitrile for 15 minutes, samples were dried in a vacuum concentrator (SpeedVac) for 15 minutes. For cysteine reduction, samples were treated with 50 µL 10 mM DTT for 45 minutes at 56 °C, followed by S-alkylation (carbamidomethylation) by incubation in 50 µL iodoacetamide (55 mM) for 30 minutes in the dark at RT. Washing steps and drying were repeated as described above. Proteins were then digested by adding 3.33 µL of Trypsin (Promega, sequencing grade 0.1 µg/µL) and 70 µL of 25 mM NH_4_HCO_3_ (~18 h at 37 °C). For extraction of peptides from the gel, samples were treated with 30 µL of 25 mM NH_4_HCO_3_ followed by 30 µL of 100% acetonitrile (both steps for 15 minutes shaking in a thermomixer). The supernatant was collected and incubated (shaking) with 30 µL of 5% formic acid (this step was repeated twice). Again, the supernatant was collected and dried in a vacuum concentrator for up to 2.5 h.

In-solution digest

Glycan profile of IgA samples were retrieved by an in-solution digest. 15 µL of purified protein (20-25 µg) samples were mixed with 30 µL of 0.1 M NH_4_HCO_3_. Cysteines were reduced with 30 µL of 15 mM DTT for 45 minutes at 56 °C and S-alkylated using 30 µL of 55 mM iodoacetamide for 30 minutes in the dark at RT. For protein precipitation samples were incubated in 500 µL of ice-cold acetone for 60 minutes at -20 °C. After centrifugation at 13000 x g for 7 minutes at 4 °C, the supernatant was removed, and samples were dried in a vacuum concentrator. The pellet was dissolved in 30 µL of 0.1 M NH_4_HCO_3_ and subjected to tryptic digestion. 5 µL of trypsin (Promega, sequencing grade 0.1 µg/µL) were added per sample and incubated at 37 °C for approximately 18 h.

| **Protein** | **Glycopeptides after enzymatic digest** | | **Molecular size (Da)** |
| --- | --- | --- | --- |
| IgG-Fc | EEQY**NST**YR | | 1189.5120 |
| Cx-Fab site | MNSLQS**NDT**AIYYCAR | Cys-CAM (Carbamidomethylation)  + 57.021 Da | 1906.8422 |
|  |  | MSO (oxidized methionine) +15.995 Da) | 1922.37 |
| IgA | GS1: SVTWSESGQ**NVT**AR | | 1521.7292 |
|  | GS2: LSLHRPALEDLLLGSEA**NLT**CTLTGLR | | 2963.5982 |
|  | GS3: TPLTA**NIT**K | | 958.5567 |
|  | GS4: LAGKPTHV**NVS**VVMAEVDGTCY | | 2347.1420 |

**Table S1**: Glycopeptides after enzymatic digest

PNGase F digestion

Aliquots of the tryptic digested samples were heat inactivated and further digested with PNGaseF (New England Biolabs) according to the manufacturer’s protocol.

Glycan analysis

Digested peptide mixtures were analyzed for their site-specific N-glycosylation profile by reversed-phase liquid chromatography-electrospray ionization mass spectrometry (LC-ESI-MS). Each peptide mixture was analyzed using a Dionex Ultimate 3000 system directly linked to a QTOF instrument (maXis 4G ETD, Bruker) equipped with the standard ESI source in the positive ion, DDA mode (= switching to MS/MS mode for eluting peaks). MS-scans were recorded (range: 150-2200 m/z) and the six highest peaks were selected for fragmentation. Instrument calibration was performed using ESI calibration mixture (Agilent). For peptide separation a Thermo BioBasic C18 separation column (5 µm particle size, 150 × 0.360 mm) was used. A gradient from 97% solvent A and 3% solvent B (Solvent A: 65 mM ammonium formiate buffer, B: 100% acetonitrile) to 32% B in 45 minutes was applied, followed by a 15 minutes gradient from 32% B to 75% B, at a flow rate of 6 µL/minute.

MS-spectra as well as the acquired MS/MS-scans of dominant precursor peaks were manually analyzed and annotated using Data Analysis 4.0 (Bruker). Glycopeptides were quantified based on signal intensity (peak height) of the highest isotope peak of each glycopeptide, assuming the isotopic pattern is the same between the analyzed ions.

Total endogenous N-glycan analysis

Plant material (1 gram) was homogenized (5% formic acid) and pepsin digested (37 °C overnight). Then the sample was purified by ion exchange chromatography (Dowex-Gel 50Wx2-400) and by gel filtration (Sephadex G25). Glycans were released by PNGase A, purified by ion exchange chromatography (Dowex-Gel 50Wx2-400), and by solid phase extraction (Phenomenex Strata C18-E). The total N-glycan profile was measured by MALDI-TOF (Strasser et al., 2004).

Glycan nomenclature according to the Consortium for Functional Glycomics ([www.functionalglycomics.org](http://www.functionalglycomics.org/)). Glycan symbols are according to the Symbol Nomenclature for Glycans (SNFG) (<https://www.ncbi.nlm.nih.gov/glycans/snfg.html>).

Antigen binding ELISA

A 20-mer peptide (P20) of the extracellular loop of human CD20 was used as an antigen (Blasco et al., 2007). 1 μg/mL of P20 (diluted in PBS buffer, pH 7.4) was coated (50 µL/well) to 96 well microplates (MicroWell™ MaxiSorp™ Merck SA M9410-1CS) overnight at 4 °C, then saturated by incubation 100 µL/well PBS-T (PBS with 0.05% Tween 20) containing 3% fat free milk powder for 1.5 h at RT. Solutions of rituximab was diluted in blocking solution in two-fold serial dilutions starting from 1000 µg/mL and applied to the coated plates (50 µL/well) incubating for 2 h at RT to obtain calibration curves. Peroxidase-conjugated goat anti-human gamma chain antibody (Merck SA I3382) was added at a dilution of 1:5000 and the plates were incubated for a further hour at RT. Every step was followed with three-time PBS-T washing. The substrate 3,3',5,5'-tetramethylbenzidine (Merk SA) was added 50 µL/well and plates were incubated ~10 minutes until the appropriate color had developed. The reaction was stopped with 2 M H_2_SO_4_ and absorbance (λ = 450 nm) with reference to 620 nm was measured with an ELISA reader (Tecan Spark® spectrophotometer).

FcγR binding by flow cytometry

FcγRIIIa (CD16A; F158 allotype) expressing TZM-bl cells were used to assess the binding affinity of Rx and Rx-RMD. Wild-type TZM-bl cells were included as a negative control to account for unspecific antibody binding. Cells were detached with Accutase, washed, and seeded into 96-well plates at 1x10^5^ cells per well. Serially diluted (1:4) antibodies were added at a starting concentration of 100 µg/mL and incubated for 45 minutes at 4 °C). Cells were washed twice with PBS and resuspended in a 1:200 dilution of a PE-labeled Fab fragment goat anti-human IgG F(ab′)2 conjugate (Jackson ImmunoResearch). After 30 minutes cells were washed twice and fixed in a 4% paraformaldehyde solution. Cells were then analyzed by flow cytometry on a NovoCyte flow cytometer (ACEA). The binding curves were generated by plotting the mean fluorescence intensity of PE-positive cells indicating receptor binding as a function of Ab concentration. Unspecific binding to wild-type cells was subtracted from binding to FcγIIIa -expressing cells. Each antibody concentration was run in duplicate. Binding experiments were repeated three times.

Acknowledgements

The COMET center: acib: Next Generation Bioproduction is funded by BMK, BMDW, SFG, Standortagentur Tirol, Government of Lower Austria und Vienna Business Agency in the framework of COMET - Competence Centers for Excellent Technologies (grant 94032). The COMET-Funding Program is managed by the Austrian Research Promotion Agency FFG. FWF Project: Austrian Science Fund (grant I 4328-B). Dr. David Montefiori and Dr. Gabriel Perez (Duke University School of Medicine, Durham, USA) for providing TZM-bl cells (contributed by through the NIH HIV Reagent Program, Division of AIDS, NIAID, NIH). Clemens Grünwald-Gruber (Core Facility Mass Spectrometry, University of Natural Resources and Life Sciences, Vienna, Austria), ~~Lin Sun (Department of Applied Genetics and Cell Biology, University of Natural Resources and Life Sciences, Vienna, Austria)~~ ~~and~~ Karin Polacsek (Department of Chemistry, University of Natural Resources and Life Sciences, Vienna, Austria) for glycan analyses ~~ELISA experiments~~ and MALDI-TOF measurements, respectively. We thank Prof. George Lomonossoff (John Innes Centre, Norwich, United Kingdom) and Plant Bioscience Limited (PBL) (Norwich, United Kingdom) for supplying the pEAQ-HT expression vector.

Supplemental References

Blasco, H., Lalmanach, G., Godat, E., Maurel, M. C., Canepa, S., Belghazi, M., Paintaud, G., Degenne, D., Chatelut, E., Cartron, G., & Le Guellec, C. (2007). Evaluation of a peptide ELISA for the detection of rituximab in serum. *J Immunol Methods*, *325*(1-2), 127-139. <https://doi.org/10.1016/j.jim.2007.06.011>

Castilho, A., Gruber, C., Thader, A., Oostenbrink, C., Pechlaner, M., Steinkellner, H., & Altmann, F. (2015). Processing of complex N-glycans in IgG Fc-region is affected by core fucosylation. *MAbs*, *7*(5), 863-870. <https://doi.org/10.1080/19420862.2015.1053683>

Goritzer, K., Maresch, D., Altmann, F., Obinger, C., & Strasser, R. (2017). Exploring Site-Specific N-Glycosylation of HEK293 and Plant-Produced Human IgA Isotypes. *J Proteome Res*, *16*(7), 2560-2570. <https://doi.org/10.1021/acs.jproteome.7b00121>

Jez, J., Antes, B., Castilho, A., Kainer, M., Wiederkum, S., Grass, J., Ruker, F., Woisetschlager, M., & Steinkellner, H. (2012). Significant impact of single N-glycan residues on the biological activity of Fc-based antibody-like fragments. *J Biol Chem*, *287*(29), 24313-24319. <https://doi.org/10.1074/jbc.M112.360701>

Li, J., Stoddard, T. J., Demorest, Z. L., Lavoie, P. O., Luo, S., Clasen, B. M., Cedrone, F., Ray, E. E., Coffman, A. P., Daulhac, A., Yabandith, A., Retterath, A. J., Mathis, L., Voytas, D. F., D'Aoust, M. A., & Zhang, F. (2016). Multiplexed, targeted gene editing in Nicotiana benthamiana for glyco-engineering and monoclonal antibody production. *Plant Biotechnol J*, *14*(2), 533-542. <https://doi.org/10.1111/pbi.12403>

Strasser, R., Altmann, F., Mach, L., Glossl, J., & Steinkellner, H. (2004). Generation of Arabidopsis thaliana plants with complex N-glycans lacking beta1,2-linked xylose and core alpha1,3-linked fucose. *FEBS Lett*, *561*(1-3), 132-136. <https://doi.org/10.1016/S0014-5793(04)00150-4>

Strasser, R., Stadlmann, J., Schahs, M., Stiegler, G., Quendler, H., Mach, L., Glossl, J., Weterings, K., Pabst, M., & Steinkellner, H. (2008). Generation of glyco-engineered Nicotiana benthamiana for the production of monoclonal antibodies with a homogeneous human-like N-glycan structure. *Plant Biotechnol J*, *6*(4), 392-402. <https://doi.org/10.1111/j.1467-7652.2008.00330.x>
