# Supplementary material for: In planta expression of active bacterial GDP‐6‐deoxy‐d‐lyxo‐4‐hexulose reductase for glycan modulation
Source: Plant Biotechnol J. 2023 Aug 8;21(10):1929–31. doi: 10.1111/pbi.14131 (PMC10502745; doi:10.1111/pbi.14131)
Supplement: Supplementary file 2 — Table S2 Quantified glyco‐profile of TSP and purified antibodies. Table S3 Quantified glyco‐profile of IgA2m1. [file PBI-21-1929-s002.pdf]

	Expression host	MM	MMX	MMF	MMXF	GnM	GnMX	GnMXF	GnGn	GnGnX	GnGnF	GnGnXF	MMX + Core Hex	GnMX + Core Hex	GnGn + Core Hex	GnGnX + Core Hex	Mannosidic	others >5%	Sum
TSP -	WT	3%	7%	0%	26%	0%	5%	12%	3%	3%	0%	26%	0%	0%	0%	0%	10%	4%	100.0%
TSP +	WT	3%	13%	0%	15%	0%	9%	6%	2%	11%	0%	15%	5%	5%	0%	3%	10%	2%	100.0%
Fcab -	WT	0%	1%	1%	23%	0%	2%	10%	10%	15%	0%	38%	0%	0%	0%	0%	0%	0%	100.0%
Fcab +	WT	0%	4%	0%	0%	0%	12%	0%	15%	68%	0%	2%	0%	0%	0%	0%	0%	0%	100.0%
2G12 -	WT	0%	0%	0%	0%	0%	0%	0%	0%	11%	0%	84%	0%	0%	0%	0%	5%	0%	100.0%
2G12 +	WT	0%	0%	0%	0%	0%	0%	0%	0%	67%	0%	17%	0%	0%	0%	0%	17%	0%	100.0%
Rx -	WT	0%	0%	0%	3%	0%	1%	6%	7%	7%	5%	66%	0%	0%	0%	0%	5%	0%	100.0%
Rx +	WT	0%	3%	0%	0%	2%	24%	0%	15%	42%	0%	7%	0%	0%	0%	0%	6%	2%	100.0%
Cx Fc -	WT	0%	0%	0%	0%	0%	0%	0%	12%	12%	0%	64%	0%	0%	0%	0%	12%	0%	100.0%
Cx Fc +	WT	0%	0%	0%	0%	0%	0%	0%	18%	39%	0%	18%	0%	0%	0%	0%	25%	0%	100.0%
Cx Fab -	WT	0%	0%	0%	0%	0%	0%	0%	0%	0%	0%	100%	0%	0%	0%	0%	0%	0%	100.0%
Cx Fab +	WT	0%	0%	0%	0%	0%	0%	0%	0%	0%	0%	64%	0%	0%	0%	26%	10%	0%	100.0%
Cx Fc -	X1	0%	0%	0%	0%	2%	0%	0%	42%	0%	34%	0%	0%	0%	0%	0%	20%	2%	100.0%
Cx Fc +	X1	0%	0%	0%	0%	5%	0%	0%	65%	4%	8%	0%	0%	0%	0%	0%	15%	3%	100.0%
Cx Fab -	X1	0%	0%	0%	0%	0%	0%	0%	0%	0%	97%	0%	0%	0%	0%	0%	3%	0%	100.0%
Cx Fab +	X1	0%	0%	0%	0%	0%	0%	0%	0%	0%	52%	0%	0%	0%	45%	0%	2%	0%	100.0%

Suppl. Table 2: Quantified glyco-profile of TSP and purified antibodies; Fcab, 2G12, rituximab (Rx), cetuximab (Cx); +/- RMD

	Expression host	MM	MMX	MMF	MMXF	GnM	GnMF	GnMX	GnMXF	GnGn	GnGnX	GnGnF	GnGnXF	MM+Core Hex	MGn + Core Hex	MMX + Core Hex	GnGn + Core Hex	GnGnX + Core Hex	GnMX + Core Hex	Mannosidic	others >5%	Sum
IgA2 GS1 -	WT	0%	0%	0%	15%	0%	0%	0%	29%	0%	0%	0%	42%	0%	0%	0%	0%	0%	0%	14%	0%	100%
IgA2 GS2 -	WT	0%	7%	0%	0%	15%	0%	0%	0%	0%	3%	0%	0%	0%	0%	0%	0%	0%	0%	61%	14%	100%
IgA2 GS3 -	WT	0%	0%	0%	26%	0%	0%	0%	27%	0%	1%	0%	35%	0%	0%	0%	0%	0%	0%	10%	0%	100%
IgA2 GS4 -	WT	0%	0%	0%	0%	0%	0%	0%	2%	0%	0%	0%	41%	0%	0%	0%	0%	0%	0%	56%	0%	100%
IgA2 GS1 +	WT	0%	0%	0%	0%	0%	0%	16%	7%	0%	33%	0%	15%	0%	0%	0%	0%	5%	2%	23%	0%	100%
IgA2 GS2 +	WT	0%	3%	0%	0%	9%	0%	33%	0%	0%	3%	0%	0%	0%	0%	0%	0%	0%	0%	40%	12%	100%
IgA2 GS3 +	WT	0%	5%	0%	8%	0%	0%	20%	11%	0%	11%	0%	17%	0%	0%	3%	0%	7%	6%	12%	0%	100%
IgA2 GS4 +	WT	0%	0%	0%	0%	0%	0%	0%	3%	0%	8%	0%	17%	0%	0%	0%	0%	9%	2%	62%	0%	100%
IgA2 GS1 -	ΔXF	9%	0%	0%	0%	22%	1%	0%	0%	44%	0%	4%	0%	0%	0%	0%	0%	0%	0%	19%	0%	100%
IgA2 GS2 -	ΔXF	4%	0%	0%	0%	17%	0%	0%	0%	16%	0%	0%	0%	0%	0%	0%	0%	0%	0%	54%	9%	100%
IgA2 GS3 -	ΔXF	13%	0%	3%	0%	23%	3%	0%	0%	35%	0%	4%	0%	0%	0%	0%	0%	0%	0%	16%	2%	100%
IgA2 GS4 -	ΔXF	0%	0%	0%	0%	4%	0%	0%	0%	26%	0%	6%	0%	0%	0%	0%	0%	0%	0%	63%	1%	100%
IgA2 GS1 +	ΔXF	8%	0%	0%	0%	23%	0%	0%	0%	52%	0%	0%	0%	0%	0%	0%	0%	0%	0%	17%	1%	100%
IgA2 GS2 +	ΔXF	3%	0%	0%	0%	19%	0%	0%	0%	13%	0%	0%	0%	0%	0%	0%	0%	0%	0%	55%	9%	100%
IgA2 GS3 +	ΔXF	0%	0%	0%	0%	30%	0%	0%	0%	49%	0%	1%	0%	0%	0%	0%	0%	0%	0%	19%	1%	100%
IgA2 GS4 +	ΔXF	0%	0%	0%	0%	6%	0%	0%	0%	33%	0%	0%	0%	0%	0%	0%	0%	0%	0%	59%	2%	100%
IgA2 GS1 + +FucT	ΔXF	3%	0%	0%	0%	6%	5%	0%	0%	9%	0%	14%	0%	2%	11%	0%	30%	0%	0%	22%	0%	100%
IgA2 GS2 + +FucT	ΔXF	3%	0%	0%	0%	18%	0%	0%	0%	18%	0%	0%	0%	0%	0%	0%	0%	0%	0%	53%	8%	100%
IgA2 GS3 + +FucT	ΔXF	0%	0%	3%	0%	5%	5%	0%	0%	3%	0%	8%	0%	11%	18%	0%	30%	0%	0%	18%	0%	100%
IgA2 GS4 + +FucT	ΔXF	0%	0%	0%	0%	0%	0%	0%	0%	5%	0%	0%	0%	0%	3%	0%	27%	0%	0%	65%	1%	100%

Suppl. Table 3: Quantified glyco-profile of IgA2m1; +/- RMD
